# MicroRNAs in biofluids are novel tools for bladder cancer screening

**DOI:** 10.18632/oncotarget.16026

**Published:** 2017-03-08

**Authors:** Xiaobing Liu, Xin Liu, Yuqi Wu, Qingjian Wu, Qingqing Wang, Zhenxing Yang, Longkun Li

**Affiliations:** ^1^ Department of Urology, Second Affiliated Hospital, Third Military Medical University, Chongqing, China

**Keywords:** bladder cancer, microRNAs, biomarker, urine, blood

## Abstract

MicroRNAs (miRNAs) are short non-coding RNAs that play important roles in basic cellular processes, including differentiation, proliferation, apoptosis and autophagy. They are also involved in various stages of tumorigenesis and play key roles in bladder cancer initiation and progression. Notably, the altered expression of miRNAs in the tumors is reflected in body fluids, including blood and urine, which opens avenues for non-invasive diagnosis and prognosis. Many studies have demonstrated that epigenetic changes extensively alter tumoral microRNA expression. The high reproducibility, specificity and sensitivity of miRNA levels in body fluids suggest their potential use as biomarkers for cancer screening and diagnosis. For example, recent technological advances have made it possible to detect miRNAs in urine for bladder cancer screening. In this review, we focus mainly on the current knowledge and future challenges for incorporating miRNAs in body fluids, like urine and blood, for making clinical diagnoses and assessing prognoses in bladder cancer.

## INTRODUCTION

Cancer is the leading cause of death and a major health problem worldwide including highly populated countries like China [1]. Bladder cancer is one of the 10 most common cancers in China that contributes to increasing morbidity and mortality. Though treatment methods such as surgery, radiotherapy, chemotherapy, and therapeutics have significantly improved the quality of life of bladder cancer patients [2], the prognosis and the 5-year survival rate is extremely poor due to late diagnosis and aggressive metastasis [3]. Early diagnosis is key for positive prognosis and to improve the survival rate [4]. Therefore, newer and more powerful methods are required to detect bladder cancer in the early stages.

Cystoscopy is a traditional method to detect bladder cancer that has benefited many patients and remains a gold standard for bladder cancer diagnosis [4]. However, it is expensive, highly invasive and causes significant discomfort to patients [5]. In recent years, alternative biological screening methods such as the bladder tumor antigen (BTA stat test), nuclear matrix protein 22(NMP22) and urinary cytology have been used [6–8]. However, these alternate methods are either highly sensitive or specific and never both.

Recently, microRNAs (miRNAs) that are short, noncoding RNA sequences of 20-22 nucleotides have emerged as novel tumor biomarker candidates [9–12]. A number of studies have demonstrated the utility of miRNAs for early stage cancer detection [10–13]. MiRNAs are involved in various stages of tumorigenesis including tumor initiation, growth and progression [14–16]. Further, miRNAs can function either as tumor suppressors (miR-15a and miR-16-1) or as oncogenes (miR-155 and members of the miR-17-92 cluster) [10]. The most promising aspect of miRNAs as molecular biomarkers is that they can be reproducibly extracted from a wide range of biological samples (such as blood plasma, urine, feces or biopsy specimen) and are generally stable and resistant to various storage conditions [17]. Also, highly sensitive and standardized techniques such as qRT-PCR, small RNA sequencing, and microarray are already available to detect and quantify miRNAs.

The potential utility of miRNAs in the diagnosis, prognosis and treatment of bladder cancer has generated enormous interest [18]. In the past decade, the number of studies related to miRNAs in urologic cancer has increased [19], highlighting the importance of miRNAs in bladder cancer [20]. Therefore, in this article, we review the importance of body fluids as a source for miRNAs as biomarkers for early diagnosis of cancer. Then, we review the development and early clinical findings regarding detection of miRNAs in blood and urine for bladder cancer screening and discuss the potential areas of improvement necessary for clinical use. In addition, we review the current available methods for miRNA detection for cancer diagnosis.

## MICRORNAS IN BIOFLUIDS AS CANCER BIOMARKERS

Fuentes-Arderiu X defined biomarker as “human or animal biological property whose *in vitro* measurement or identification is useful for the prevention, diagnosis, prognosis, treatment, and follow-up of human or animal diseases, and for their understanding” [21]. Many studies using The Cancer Genome Atlas (TCGA) data revealed that dysregulated miRNA expression profiles in cancer tissues was associated with tumor state, grade, size, aggressiveness and metastasis [22–24]. The current goal of precision medicine is to achieve personalized health care by classifying patients into distinct molecular groups accompanied with accurate prediction of disease risk to enable early intervention with the most suitable therapeutic approach to achieve better outcomes and avoid unnecessary treatment with adverse effects [25]. Therefore, miRNAs are good candidates as precision molecular markers for diagnosis, therapy choice and response surveillance.

**Figure 1:**
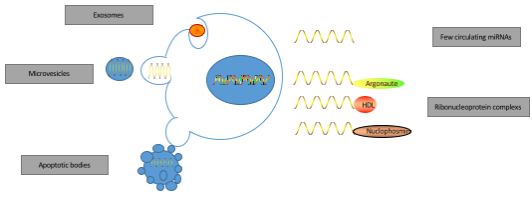
The origin of miRNAs in biofluids

MiRNAs are useful biomarkers because changes in miRNA in the tumors can be non-invasively detected in body fluids like blood and urine [26]. MiRNAs are secreted into body fluids by tumor cells via specific regulated pathways (Figure 1). The miRNAs can be detected in body fluids as either free circulating miRNAs or those that are bound to ribonucleoprotein complexes and encapsulated in extracellular vesicles [27–31]. The main protein binding partners of miRNAs are Argonaut proteins, high density lipoproteins (HDL) and nucleophosmins [27–29]. Also, miRNAs are secreted in extracellular vesicles such as exosomes, microvesicles or apoptotic bodies. Exosomes are multivesicular bodies that fuse with the plasma membrane; microvesicles are derived from outward budding of the cell membrane; and apoptotic bodies are formed during late stages of apoptosis via membrane blebbing that contain nuclear components and cytoplasm [31]. The miRNAs that are part of complex proteins and lipids or encapsulated in extracellular vesicles are protected from degradation and are stable in body fluids [32, 33]. The most extensively studied miRNAs are exosome-encapsulated miRNAs. It is observed that miRNA content in the secreting cells and the exosomes is different suggesting selective packaging of miRNAs into exosomes [32]. Further, enhanced exosome-encapsulated miRNAs is observed in metastatic cancer cells, which signifies their role in tumorigenesis [34]. Studies also indicate that miRNAs enable cell-cell communication, although the function and mechanism is still being investigated and is unclear [12].

MiRNAs can be easily screened and precisely quantified by a variety of standard methods like qRT-PCR, insitu hybridization, enzymatic luminescence miRNA assay, microarray, or next-generation sequencing [35], which can be applied clinically to detect tumors at an early stage. The miRNAs are optimal biomarker candidates because they can be reproducibly isolated from a wide range of biological fluids and are generally stable and resistant to various storage conditions [17]. However, the development of a biomarker assay for the clinic is a multistage process and includes (1) identification of potential markers in discovery phase; (2) development of specificity and sensitivity assays, and (3) extensive validation steps. Currently, there are many methodological pitfalls that need to be addressed in order for miRNAs from biofluids to be used for clinical applications. These include improving and standardizing methods of sample collection, storage, RNA isolation, sequencing and data evaluation [36–40]. In spite of these challenges that require rigorous studies, miRNAs in body fluids have enormous potential as novel diagnostic and prognostic markers for cancer screening.

## MICRORNA IN BODY FLUIDS AND BLADDER CANCER

In most cases, bladder cancer has metastasized at diagnosis [41]. Bladder cancer is classified into two different types based on the pathology, namely, non-muscle invasive bladder cancer (NMIBC) that is confined to the mucosa or submucosa, and muscle invasive bladder cancer (MBIC) that has invaded the muscle. The European Association of Urology (EAU) guidelines on NMBIC define three main purposes in applying new molecular biomarkers: (1) screening of the population at risk of developing bladder cancer; (2) evaluation of patients with symptoms suspicious for bladder cancer; and (3) facilitate surveillance of patients with bladder cancer to reduce the number of cystoscopies undertaken [42]. Therefore, in addition to the diagnosis of cancer, the surveillance of recurrence and progression from NMIBC to MIBC are critical. In regard to MIBC, different therapeutic options can alter progression and metastasis that are evaluated by disease free survival (DFS), cancer specific survival and overall survival (OS). Also, both NMIBC and MIBC have different molecular characteristics [43]. There are several methods to diagnose bladder cancer. Since the standard diagnostic tool, cystoscopy, has low diagnostic sensitivity in detecting low-grade bladder cancer in addition to being inconvenient and costly for patients, numerous non-invasive urine based tests BTA stat test, nuclear matrix protein 22(NMP22) and urinary cytology have been used [6–8]. However, these alternate methods are either highly sensitive or specific and never both. However, the dysregulation of miRNAs in bladder cancer tissue is also manifest in urine and blood demonstrating their advantage as new biomarkers for bladder cancer [44]. Therefore, different miRNA patterns in the body fluids may reflect the progressive nature of the bladder cancer that could potentially be used for diagnostic and prognostic purposes in the clinic [45].

## MICRORNAS IN URINE SAMPLES

In 2010, Hanke et al first reported miRNAs in urine samples as a diagnosis tool for bladder cancer [46]. Since then, a number of single-center, case-control studies have reported in this area [46–66]. Only one study has focused on miRNAs for surveillance in bladder cancer which using a panel of 12 miRNAs shows promise for detection of tumor recurrence in the surveillance of bladder cancer [64]. And multi-center, prospective cohort studies have not been reported so far. Yet, miRNAs in biological fluids for bladder cancer hold enormous promise as biomarkers due to their high specificity and sensitivity [47, 49, 50, 67] (Table 1). Increasing studies have reported on the potential of miRNAs as prognostic markers, although specific miRNAs have not yet been authenticated [52, 63, 64]. Further, studies have implied that combination of miRNAs enhance the specificity and sensitivity of diagnostic assays than single miRNAs alone [53, 54, 64, 68].

**Table 1:** Studies regarding miRNAs in urine as potential biomarkers in bladder cancer

Study	Year	Sample	Results	BC/Cont(n	clinically relevated findings	Reference
Hanke *et al*.	2010	Whole urine	Upregulate: miR-126,miR182	29//11	AUC=0.768, DS=72%, DSp-83%	46
Yamada *et al*.	2011	Urine sediment	Upregulate: miR96, miR-183	100/74	AUC=0.831/0.817, DS=71/74%, DSp=79/77%	47
Miah *et al*.	2012	Urine sediment	Upregulate: miR-15b, miR-1224-3p; downregulate: miR-135	68/53	AUC=0.86, DS=94.1%, DSp=51%	48
Puerta-Gil *et a*	2012	Urine (not defined)	Upregulate: miR-222, miR-452; downregulate: miR-143	37/57	AUC=0.718, AUC=0.848	49
Snowdon *et al*	2012	Whole-urine	Upregulate: miR-126; downregulate: miR-125b	8/5	without data	55
Wang *et al*.	2012	Urine sediment and superna	Upregulate: miR-141, miR200a/b/c, miR-429	51/24	AUC=0.706-0.804, DS=100%, DSp=53%	50
Yun *et al*.	2012	Urine supernatant	Upregulate: miR-145, miR-200a	207/144	AUC=0.729 and 0.790, DS=78% and 84%, DSp=61% and 61	51
Kim *et al*.	2013	Urine supernatant	Upregulate: miR-214	138/144	without data	52
Mengual *et al*.	2013	Urine sediment	Upregulate: miR-18a, miR-25, miR-187, miR92a; downregulate: miR-140-5p, miR-142-3p, mi	151/121	AUC=0.92, DS-85%, DSp=87%	53
Shimizu *et al*.	2013	Urine supernatant	Upregulate: miR-9-3, miR-142-2/3, miR-137	86/20	AUC=0.91	54
Tolle *et al*.	2013	Whole urine	Upregulate: miR-520e, miR-618, miR-122-5p	36/19	AUC=0.679-0.764	56
Zhang *et al*.	2014	Urine supernatant	Upregulate: miR-99a, miR-125b	50/21	AUC=0.876, DS=79%, DSp=88%	57
Zhou *et al*.	2014	Urine supernatant	Upregulate: miR-106b	112/78	AUC=0.802, DS=76.8%, DSp=72.4%	58
Eissa *et al*.	2015	Urine sediment	Upregulate: miR-96	94/90	AUC=0.822, DS=76.8%, DSp=88.9%	59
Eissa *et al*.	2015	Urine sediment	Upregulate: miR-210, miR-96	94/56	AUC=0.933, DS=100%, DSp=89.5%	60
Liu *et al*.	2015	Urine sediment	Upregulate: miR-141, miR-200b	78/54	AUC=0.749	61
Long *et al*.	2015	Urine supernatant	Upregulate: let-7b, miR-15a, miR-21, miR-26a, miR-93, miR-101, miR-200c, miR-940	85/45	AUC=0.858, DS=70%, DSp=84%	62
Wang *et al*.	2015	Urine supernatant	Upregulate: miR-214,	292/169	AUC=0.838, DS=90.5%, DSp=65.6%	63
Spare *et al*.	2016	Whole urine	Upregulate: miR-16, miR-21, miR-34a, miR-200c, miR-205, miR-211	60/21	AUC=0.74, DS=88%, DSp=48%	64
Urquidi *et al*.	2016	Uriene(not defined)	25-miRNA model	88/118	AUC=0.982	66

I order to translate the potential indicated by the preliminary studies, potential biomarker miRNAs need to be validated using large patient cohorts and multi-center studies. The current data is not amenable for validation because of multiple varying factors such as different objectives of each study, the heterogeneity of study cohorts and variation in the source and preparation of urine test materials (whole urine, sediment urine, urine supernatant, and urine exosome). Also, lack of standard protocol in processing the samples inspite of commercially available kits (Norgen, Exiqon, Qiagen) is a problem [57]. Further, there is great variation in the miRNAs or miRNA combinations that are focused in each study due to investigator's interest or the available tumor samples. Such a methodology is not optimal to standardize miRNA biomarkers in urine for bladder cancer screening. Another important factor that needs to be considered for clinical purposes is that miRNAs released from cultured cells are the basis of most investigations and may not be representative of those found in the biological fluids or primary tumors. Therefore, comprehensive and standardized methods of biomarker detection are necessary that need to be applied to such studies worldwide. The most widely accepted protocol is preliminary investigation based on genome-wide discovery methods followed by sequencing or microarray investigations and analysis, and subsequent validation of candidate miRNAs by qRT-PCR [62, 68, 69]. Importantly, only two studies (Sapre *et al* [64] and Eissa *et al* [60]) followed the EAU guidelines while evaluating the diagnostic specificity and sensitivity of the miRNAs that mandates direct comparison of the performance of candidate miRNAs with the gold standard of cytology or other urine tests. Eissa *et al* screened bladder cancer patients with negative cytoscopy results by combining cytology results with expression of miR-96 and miR-210 and achieved an AUC value of 0.933 [60]. Sapre *et al* used the integrative approach with a panel of 12 miRNAs that reduced cystoscopy rate by 30% through enhancing diagnostic sensitivity and specificity to 88% and 48%, respectively [64]. Currently, further study is necessary in this area as according to Standards for Reporting Diagnostic Accuracy (STARD) guidelines, the new method needs to be compared with the gold standard and the data published [70]. In addition to these issues, several studies have associated miRNAs with hemolysis [71, 72]. Since hematuria is a critical symptom of bladder cancer [42], miRNAs are released from the erythrocytes and these must be excluded during bladder cancer screening. Therefore, it is also important to identify miRNAs that are bonafide and cancer-specific versus those that are due to hematuria.

## MICRORNAS IN SERUM AND PLASMA SAMPLES

Table 2 shows the studies reporting miRNA in blood samples suggesting a similar trend to urine [56, 68, 69, 73–81]. Although deregulated miRNAs were previously reported in bladder cancer tissues, the first report on miRNA screening in blood samples was reported in 2013 by Adam *et al* [75]. In 2015, Du *et al* [68] and Jaing *et al* [69] used genome-wide, array-based profiling of large samples and validation cohorts that critically analyzed the diagnostic and prognostic potential of circulating miRNAs. Du *et al* used two-phase validation method and showed that two miRNAs from a list of eight miRNAs previously identified in the discovery phase,namely, miR-497 and miR-663b demonstrated enhanced sensitivity and specificity with an AUC value of 0.711 [69]. Jiang *et al* used a similar method to achieve an AUC value of 0.899 for a panel of six miRNAs that differentiate patients with NMIBC and MIBC [68]. In both studies, the miRNA combinations demonstrated the potential to predict tumor recurrence.

**Table 2:** Studies regarding miRNAs in blood as potential biomarkers in bladder cancer

Study	Year	Sample	Results	BC/Cont(n	clinically relevated findings	Reference
Tolle *et al*.	2013	Whole-bloo	Upregulate: miR-26a-5p, mir-144-5p, miR-374-5p	38/20	AUC=0.774-0.824	56
Adam *et al*.	2013	Plasma	No different	20/18	no difference	75
Scheffer *et a*	2014	Serum	miR-141,miR-639	126/105	No different levels between study gr	73
Feng *et al*.	2014	Plasma	Upregulate: miR-99a	50/50	without data	80
Feng *et al*.	2014	Plasma	Upregulate: miR-19a	50/50	without data	76
Jiang *et al*.	2015	Serum	Upregulate: miR-152; downregulate: miR-1486-3p, miR-3187-3p, miR-15b-5p, miR-27a-	120/120	AUC=0.899, DS=80%, DSp=89%	68
Kreebel *et al*.	2015	Serum	Upregulate: miR-1141	34/34	AUC=0.726, DS=70.5%, DSp=73.5	74
Du *et al*.	2015	Plasma	Upregulate: miR-663b; downregulate: miR-497	165/175	AUC=0.711, DS=69.7%, DSp=69.6	69
Yang *et al*.	2015	Serum	Upregulate: miR-210	168/104	without data	79
Tao *et al.*	2015	serum	13-miRNA model	46/30	AUC>0.8	81
Motawi *et al*.	2016	Plasma	downregulate: miR-92a, miR-100, miR-143	70/62	AUC=0.926	78

Based on the preliminary studies discussed above, miRNAs in urine and blood can be potential biomarkers for bladder cancer and need to be investigated further. Therefore, well-designed, multi-center, prospective studies with optimal isolation conditions, data analysis, study design and miRNA panels are of critical importance to evaluate clinical screening of miRNAs in urine and blood for bladder cancer.

## COMMON METHODS FOR MICRORNA DETECTION

The challenges in detecting miRNAs in body fluids for clinical evaluations include reproducibility and methodological pitfalls. The critical factors that affect optimal miRNA measurements include sample collection methods, processing and storage conditions, RNA isolation technique, quality control, quantification principle and data evaluation methods. Sample collection is a very important step that influences the stability of the final results. Currently, there are no standardized conditions described for this step. It is important that criteria like age, ethnic group, gender and prior treatment be considered [82]. The three generally used miRNA isolation methods are miRNeasy kit, TRIzol and mirVANA [83], which have slight differences that may be a source of bias. Therefore, researchers should choose appropriate technology to detect miRNAs in body fluids that would ensure stability and reproducibility [82]. In most cases, Students t-test is used to analyze the statistical significance of the differences in samples to determine their clinical relevance [84].

For quantitative and qualitative evaluation of miRNAs, various methods like Northern blotting, microarray technology, luminescence-based assays, electrochemical assays and *in situ* hybridization [85]. In most cases, the assays are based on Watson-Crick base-pairing between complementary chains of nucleotides and require hybridization between the nuclei acid probe and target miRNA. The miRNA expression can vary based on the detection methods such as microarray and qRT-PCR. For clinical applications, acquisition of highly reproducible data is necessary to produce a reliable profile. Table 3 compares the application, advantages, disadvantages and improvements of various detection methods including Northern blot analysis, bioluminescence, qRT-PCR, fluorescence correlation spectroscopy, *in situ* hybridization and microarray [86–102].

**Table 3:** Characteristics of common methods used in miRNA diagnosis

Methods	Advantage	Limitation	Reforence
RT-RCR	Easy to operate and high sensi	Low thoughput, Expen	91-93
Microarray	High throughput	Lack of quantitative dat	99-102
Nortern blot analysis	Gold standard, High specificity	Poor sensitivity	86-89
Bioluminescence	High sensitivity	Complex steps	90
In situ hybridization	High speicificity	Low thoughput, Expen	95-98
Fluorescence correlation spectro	High sensitivity	Special equipment	94

For clinical applications, laboratory scientists, bioinformaticians, clinicians and statisticians need to ensure reliable and reproducible data. Thus, standardizing highly reliable protocols to obtain miRNA samples and highly sensitive methods to accurately and reproducibly evaluate the miRNAs is critical for establishing them as biomarkers.

## FUTURE PERSPECTIVES

Currently, studies indicate that non-invasive detection of circulating miRNAs from body fluids of bladder cancer patients reflect the dysregulation in bladder cancer patients at different cancer stages. Increasing studies have investigated circulating miRNAs in bladder cancer in the past five years and next-generation sequencing has become more affordable and faster enabling rapid sequencing of biofluids in large-scale, multicenter studies. Notably, a number of studies indicated that the miRNAs changes in tissue are not always consistent in blood and urine, highlighting that secretion pathway exists from tissue to biofluids [19, 77]. However, systematic multicenter and prospective cohort studies evaluating specific miRNAs that have been studied in tissue samples is missing in the biofluids. The currently available data is unreliable due to differences in methodology in various studies and heterogeneity of tested biofluids. Furthermore, in the absence of complementary studies, potentially important miRNAs may still be undiscovered and currently the clinical application based on available data is limited. Further, since the miRNA secretion pathway influences the stability of miRNAs in the biofluids, exosomal miRNA and miRNA bound in ribonucleoprotein complexes are stable and most suitable for the sensitive and robust marker detection, whereas free circulating miRNAs and miRNAs bound to proteins or packaged in vesicles are unsuitable due to their limited stability.

Currently, there is no standard consensus regarding methods for biofluids preparation and miRNA sequencing. In general multiple sources of blood (plasma, serum and whole blood) and urine (supernatant, sediment and whole urine) are randomly used for miRNA isolation. Further, factors such as hematuria and hemolysis that influence the reliability of the outcomes need to be considered to mitigate the bias. Next generation sequencing and digital PCR are more accurate and sensitive and need to be considered than the conventional methods such as microarrays and qRT-PCR that have inherent weaknesses and inconsistencies [103].

Further, multicenter, prospective cohort studies with stringent test conditions are necessary to validate circulating miRNAs. Also, other specific clinical needs such as surveillance markers need to be addressed to reduce the need of the invasive cystoscopy for bladder cancer patients. From the statistical standpoint, further studies that incorporate evaluation using multivariate models to evaluate miRNA combinations is necessary. If all these aspects are considered and rigorously evaluated, miRNAs and miRNA combinations hold enormous promise as diagnostic, prognostic, and predictive markers.

## CONCLUSIONS

To conclude, circulating miRNAs in biofluids are promising markers for potential non-invasive bladder cancer screening. Among the various challenges ahead, overcoming the methodological bias by highly standardized and sensitive technologies and rigorous multiple center, prospective cohort studies are necessary to identify and establish circulating miRNAs as biomarkers for bladder cancer for clinical applications in the near future.
